# Selective inhibition of receptor activator of NF-κB ligand (RANKL) in hematopoietic cells improves outcome after experimental myocardial infarction

**DOI:** 10.1007/s00109-018-1641-x

**Published:** 2018-05-08

**Authors:** Svetlana Slavic, Olena Andrukhova, Kristopher Ford, Stephan Handschuh, Nejla Latic, Ursula Reichart, Soleman Sasgary, Claudia Bergow, Lorenz C. Hofbauer, Paul J. Kostenuik, Reinhold G. Erben

**Affiliations:** 1 Institute of Physiology, Pathophysiology and Biophysics, Department of Biomedical Research, University of Veterinary Medicine Vienna, Veterinaerplatz 1, 1210 Vienna, Austria; 2 VetCore, University of Veterinary Medicine Vienna, Vienna, Austria; 3 Division of Endocrinology, Diabetes, and Bone Diseases, Department of Medicine III and Center for Healthy Aging, Technische Universität Dresden, Dresden, Germany; 4 Amgen Inc., Thousand Oaks, CA USA; 5 Present Address: Phylon Pharma Services, Newbury Park, CA USA

**Keywords:** Myocardial infarction, RANKL, Inflammation, Osteoprotegerin

## Abstract

**Abstract:**

The RANK (receptor activator of nuclear factor κB)/RANKL (RANK ligand)/OPG (osteoprotegerin) axis is activated after myocardial infarction (MI), but its pathophysiological role is not well understood. Here, we investigated how global and cell compartment-selective inhibition of RANKL affects cardiac function and remodeling after MI in mice. Global RANKL inhibition was achieved by treatment of human RANKL knock-in (huRANKL-KI) mice with the monoclonal antibody AMG161. huRANKL-KI mice express a chimeric RANKL protein wherein part of the RANKL molecule is humanized. AMG161 inhibits human and chimeric but not murine RANKL. To dissect the pathophysiological role of RANKL derived from hematopoietic and mesenchymal cells, we selectively exchanged the hematopoietic cell compartment by lethal irradiation and across-genotype bone marrow transplantation between wild-type and huRANKL-KI mice, exploiting the specificity of AMG161. After permanent coronary artery ligation, mice were injected with AMG161 or an isotype control antibody over 4 weeks post-MI. MI increased RANKL expression mainly in cardiomyocytes and scar-infiltrating cells 4 weeks after MI. Only inhibition of RANKL derived from hematopoietic cellular sources, but not global or mesenchymal RANKL inhibition, improved post-infarct survival and cardiac function. Mechanistically, hematopoietic RANKL inhibition reduced expression of the pro-inflammatory cytokine IL-1ß in the cardiac cellular infiltrate. In conclusion, inhibition of RANKL derived from hematopoietic cellular sources is beneficial to maintain post-ischemic cardiac function by reduction of pro-inflammatory cytokine production.

**Key messages:**

- Experimental myocardial infarction (MI) augments cardiac RANKL expression in mice.
- RANKL expression is increased in cardiomyocytes and scar-infiltrating cells after MI.
- Global or mesenchymal cell RANKL inhibition has no influence on cardiac function after MI.
- Inhibition of RANKL derived from hematopoietic cells improves heart function post-MI.
- Hematopoietic RANKL inhibition reduces pro-inflammatory cytokines in scar-infiltrating cells.

**Electronic supplementary material:**

The online version of this article (10.1007/s00109-018-1641-x) contains supplementary material, which is available to authorized users.

## Introduction

Improvements in the prevention and treatment of cardiovascular diseases have significantly changed the epidemiology of myocardial infarction (MI). Whereas early survival has been improved and the severity of infarctions has declined progressively, the overall incidence of MI and the long-term survival has remained constant [1, 2]. Long-term mortality after MI is still high, partly due to progression of the disease to heart failure (HF), a condition which requires improvement in treatment options. Thus, a better understanding of the molecular mechanisms involved in HF progression is necessary for development of novel therapeutic strategies to prevent heart failure post-MI. In this context, an increasing body of evidence indicates that signaling through the RANK (receptor activator of nuclear factor κB)/RANKL (RANK ligand)/OPG (osteoprotegerin) axis might be involved in the pathophysiology of cardiovascular diseases. RANKL was first discovered as a cytokine which drives macrophage maturation to osteoclasts through its signaling receptor RANK, while OPG inhibits RANKL signaling acting as a soluble decoy receptor of RANKL [3]. Besides its role in bone remodeling, baseline levels of soluble RANKL predict the risk of cardiovascular events such as myocardial infarction and stroke [4]. Furthermore, it was suggested that RANKL may be important for destabilizing atherosclerotic plaques [4, 5], whereas OPG may prevent blood vessel calcification in atherosclerosis [6]. Notably, the myocardial OPG/RANKL ratio is significantly lower in clinical and experimental heart failure, due to disproportionally enhanced RANKL expression [7]. However, it is currently not known which exact role RANKL plays during the post-ischemic myocardial healing and during transition to HF.

The complex NF-κB signaling initiated by binding of RANKL to its receptor RANK can be, depending of the cellular context, detrimental or protective [8]. It was suggested that RANKL promotes myocardial inflammation during acute cardiac overload [9] and favors adverse remodeling by matrix degradation after acute myocardial infarction [7]. In addition, RANKL was reported to act as a pro-inflammatory cytokine on hepatocytes as shown by the finding that hepatic RANKL deletion counteracted hepatic insulin resistance by reducing NF-κB signaling [10]. On the other hand, increased RANKL signaling reduced the infarct size in stroke and hepatic ischemia, by acting in an anti-inflammatory and pro-cell survival manner [11, 12].

Here, we aimed to investigate the effect of RANKL inhibition on post-ischemic cardiac function and structure. We inhibited RANKL using the human monoclonal IgG1 anti-RANKL antibody AMG161. AMG161 selectively inhibits human but not murine RANKL, by binding to a peptide sequence encoded by exon 5 of the human RANKL gene. To block RANKL in vivo, we used a transgenic mouse line (human RANKL knock-in, huRANKL-KI) where exon 5 of the murine RANKL gene was replaced by the human sequence [13]. The chimeric RANKL protein expressed by huRANKL-KI mice is capable of inducing bone resorption, while being fully inhibited by AMG161 [13]. Based on our previous finding that only endothelial and hematopoietic but not stromal precursors engraft after transplantation of unfractionated bone marrow into lethally irradiated rats [14] and mice [15] and the fact that AMG161 exclusively inhibits human but not murine RANKL, we used a reconstitution model in which we selectively exchanged the hematopoietic cell compartment to dissect the pathophysiological role of RANKL derived from hematopoietic and mesenchymal cellular sources in the development of cardiac dysfunction after ischemia. We found that inhibition of RANKL derived from hematopoietic cellular sources had beneficial effects on cardiac function after MI.

## Methods

### Animals

All animal procedures were undertaken in accordance with current guidelines for animal care and welfare and were approved by the Ethical Committees of the University of Veterinary Medicine Vienna and of the Austrian Federal Ministry of Science, Research and Economy. All animals were kept in groups of two to seven at 22–24 °C and a 12-h light/12-h dark cycle with free access to tap water and a commercial rodent diet (Sniff™). The generation of the huRANKL-KI transgenic mouse line was described in detail elsewhere [13]. huRANKL-KI mice were obtained from Amgen Inc. and were backcrossed to C57BL/6 genetic background for a minimum of six generations. Heterozygous mice were bred, and the resulting wild-type (wt) and homozygous huRANKL-KI animals were genotyped from tail biopsies by PCR analysis of genomic DNA.

### Lethal irradiation and bone marrow transplantation

Male wt and huRANKL-KI mice at 6 to 8 weeks of age were used as bone marrow (BM) donors. Mice were killed by exsanguination from the abdominal vena cava under ketamine/xylazine anesthesia (70/7 mg/kg i.p.). Unfractionated BM was isolated from femora, tibias, and humeri by centrifugation at 800×*g* for 5 min at room temperature. The cells suspended in PBS were filtered through a 40-μm cell strainer and were re-suspended in PBS to contain 4 × 10^7^ cells per milliliter.

Male 16-week-old wt and transgenic huRANKL-KI recipient mice were lethally irradiated with a single dose of 10 Gray, using a linear accelerator (6MV, Primus, Siemens). Four hours after the irradiation, 4 × 10^6^ of freshly prepared unfractionated BM cells were injected into a lateral tail vein of the recipient mice. Irradiated wt mice received the BM cells of huRANKL-KI donors, and irradiated huRANKL-KI mice received BM cells of wt donors. We previously demonstrated that this protocol efficiently reconstitutes cells of the hematopoietic origin with a chimerism greater than 90% as analyzed by flow cytometry, 4 weeks post-transplantation [15]. To avoid infections during the aplastic phase, irradiated animals were daily subcutaneously treated with an antibiotic (enrofloxacin, 10 mg/kg) over 7 days. Animals were left to recover for 4 weeks before they were subjected to sham surgery or to myocardial infarction.

### Myocardial infarction

Twenty-week-old male huRANKL-KI and wt mice were anesthetized with ketamine/medetomidine (100/0.25 mg/kg i.p.) anesthesia. Endotracheal intubation was performed after disappearance of the paw pinch reflex. Animals were ventilated with a tidal volume of 200 μL and a frequency of 210 breathing cycles per minute using a small animal ventilator (MiniVentTyp 845, Hugo Sachs Elektronik-Harvard Apparatus GmbH). Permanent ligation of the left descending coronary artery was performed after a left lateral thoracotomy. Analgesic (buprenorphine 0.25 mg/kg s.c.) and antibiotic (enrofloxacin, 10 mg/kg s.c.) were injected for 4 and 5 days, respectively. Sham animals underwent the same procedure but without the arterial ligation.

Animals were killed 4 weeks after MI by exsanguination from the abdominal vena cava under ketamine/xylazine anesthesia (70/7 mg/kg i.p.). This time point was chosen in order to be able to document a robust decline in cardiac functional parameters after MI. Serum samples, hearts, aortas, bones (femora, L4 vertebra), and bone marrow were flash frozen and stored at − 80 °C until assayed, or processed for histological analysis.

### Global and compartment-selective RANKL inhibition by AMG161

**Fig. 1:**
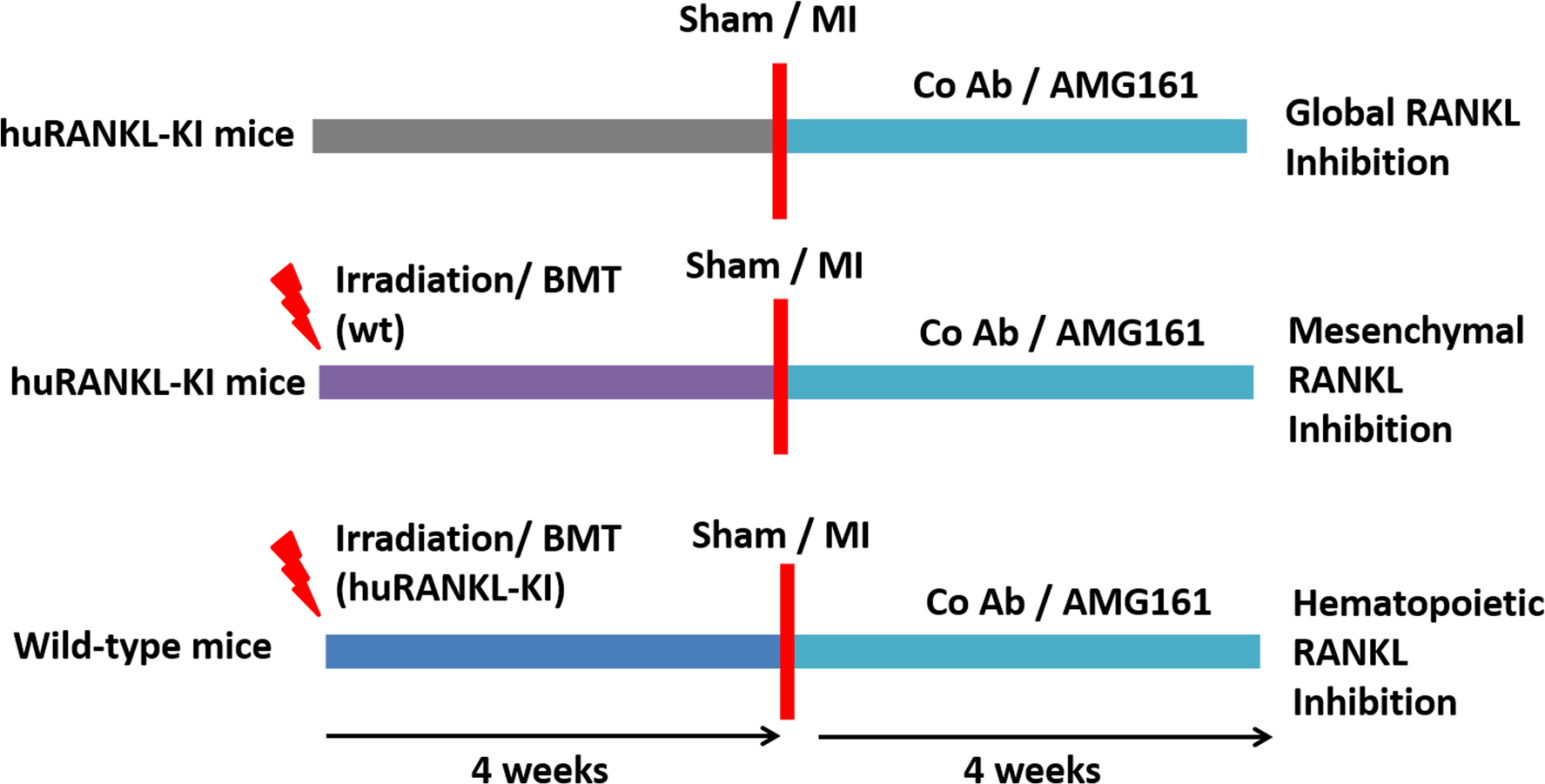
Study design of global and compartment-selective RANKL inhibition. Irradiation and bone marrow transplantation (BMT) were performed in 16-week-old mice. Sham surgery or myocardial infarction (MI) were performed in 20-week-old mice

Animals were randomized in a blinded fashion to the treatment with the anti-RANKL antibody AMG161 or an isotype control antibody (anti-keyhole limpet hemocyanin, KLH). Both AMG161 and the control antibody are humanized IgG1 antibodies. All antibodies were dissolved in A5su buffer containing 0.004% Tween 20 and were kindly provided by Amgen Inc. AMG161 or control antibody treatment (both at 10 mg/kg) started post-operatively and was continued twice weekly for the duration of 4 weeks. Global inhibition of RANKL was achieved by treatment of homozygous huRANKL-KI mice with AMG161. To inhibit RANKL derived from cells of hematopoietic origin, wt mice were lethally irradiated, reconstituted with bone marrow from homozygous huRANKL-KI donors, and subsequently treated with AMG161. Vice versa, administration of AMG161 to lethally irradiated homozygous huRANKL-KI mice which were previously reconstituted with bone marrow from wt mice, blocked RANKL from mesenchymal cell sources. The study design is graphically presented in Fig. 1.

### Transthoracic Doppler echocardiography

Left ventricular (LV) function was non-invasively assessed 7 days and 3 weeks after MI in mice under isofluorane anesthesia using a 14-MHz linear transducer (Siemens S2000). Short-axis M-mode recordings of LV were obtained from a left parasternal acoustic window. LV dimensions in systole and diastole were measured through the largest diameter of the LV at the level of papillary muscles, and fractional shortening (FS) was calculated. Diastolic LV function was evaluated using the pulsed-wave Doppler recording of trans-mitral blood flow velocities in the apical four-chamber view. A minimum of four cardiac cycles were averaged for each measured parameter.

### Central arterial and cardiac pressure measurement

Pressure was assessed using a SPR-671NR pressure catheter (1.4F, Millar Instruments, Houston, TX, USA). The catheter was inserted into the ascending aorta via the carotid artery and central arterial pressure was measured under 1.0% isofluorane anesthesia. Thereafter, the catheter was advanced into the LV for measurement of cardiac pressure parameters. Pressure was recorded over 5 min and traces were analyzed using LabChart 7 software and a blood pressure module.

### Infarct size measurement

Hearts excised at necropsy were washed from blood with PBS and fixed using 40% ethanol for at least 48 h. Dehydrated and paraffin-embedded hearts were sectioned at 5 μm thickness and were stained with Masson’s trichrome staining to visualize muscle and infarct tissue. At least nine standardized sections between basis and apex of the heart were evaluated using AMIRA software. Infarct area was calculated as percentage of total LV area.

### Gene expression analysis

Total RNA from LV tissue was isolated using TRI Reagent® Solution (Invitrogen). The concentration, purity, and quality were determined spectrophotometrically (NanoDrop 2000; Thermo Scientific) and by 2100 Bioanalyzer (Agilent Technologies). One microgram of RNA was reverse transcribed (High Capacity cNDA Reverse Transcription Kit; Applied Biosciences). Quantitative RT-PCR was performed on a Vii7 device (Applied Biosystems®) using the 5× Hot Firepol® Eva Green kit (Solis Biodyne). To exclude amplification of the genomic DNA, primers were designed as exon spanning and their sequence is available upon request. A product melting curve analysis was performed to exclude primer dimerization and nonspecific amplification. All samples were measured in duplicate and expression values were normalized to ornithine decarboxylase antizyme-1 (*Oaz1*) mRNA.

### Serum and urine biochemistry

Serum phosphorus, calcium, alkaline phosphatase, and urinary creatinine were analyzed using a Cobas c111 analyzer (Roche). Total urinary deoxypyridinoline (DPD) concentrations were assessed by a commercially available ELISA (MicroVue DPD EIA kit, Quidel) after acid hydrolysis.

### pQCT analysis

Bone specimens were collected at necropsy and stored in 70% ethanol until analysis of mineral density using a XCT Research M+ pQCT device (Stratec Medizintechnik).

### Immunohistochemistry

Antigen retrieval was performed by heating the de-paraffinized cardiac sections to 100 °C for 15 min in citrate buffer (pH 6). Sections were then treated with 0.1% Triton X-100 for 5 min at room temperature to permeabilize cell membranes, and for further 30 min with blocking solution containing 10% goat serum and 0.02% Triton X in PBS to prevent unspecific antibody binding. Primary antibody against RANKL (rabbit polyclonal IgG, 1:200 in blocking solution, Santa Cruz Biotechnology), IL-1ß (goat polyclonal, 1:500 in blocking solution, R&D Systems), and CD68 (rat monoclonal, 1:100, Bio Rad) were incubated overnight at 4 °C. After washing, secondary biotinylated antibodies (Vector) were added and incubated for 60 min at room temperature. Signal was developed by incubation with streptavidin-peroxidase (Vector) followed by 3-amino-9-ethyl carbazol (AEC) or DAB staining.

### Immunofluorescence

Sections were prepared for staining as described for immunohistochemistry. For co-staining of RANKL and CD3, primary antibody against RANKL (rabbit polyclonal IgG, 1:100 in blocking solution, Santa Cruz Biotechnology) and anti-CD3 (Monoclonal Rat IgG, 1:70 in blocking solution, R&D Systems) were incubated overnight at 4 °C. After washing, secondary anti-rabbit Alexa Fluor 555 (Molecular Probes, 1:500) and anti-rat Alexa Fluor 594 (Thermo Fisher, 1:500) were incubated at room temperature for 60 min. Co-immunostaining of RANKL and cardiac troponin T was performed in two steps. Firstly, primary anti-RANKL antibody was incubated overnight as described above. Subsequently, biotinylated secondary anti-rabbit antibody (Vector, 1:400) was incubated at room temperature for 1 h, followed by incubation with Streptavidin-Alexa 546 conjugate (Molecular Probes) for 30 min. After washing, primary rabbit anti-mouse cardiac troponin T antibody (St John’s Laboratory) and secondary anti-rabbit Alexa Fluor 647 (Thermo Fisher) antibodies were sequentially incubated for 1 h at room temperature. Immunostainings where primary antibodies were omitted served as negative control. Co-immunostaining of IL-1ß and CD45 was performed by incubating primary antibodies (rabbit anti-mouse IL1ß, Abcam, 1:200; rat anti-moue CD45, BD Pharmingen, 1:40) overnight at 4 °C followed by incubation with secondary anti-rabbit Alexa Fluor 555 and anti-rat Alexa Fluor 594 at room temperature for 60 min. Co-staining of IL-1ß and troponin T was performed in a two-step reaction as above described. Nuclei were stained with DAPI (4′,6-diamidino-2-phenylindole). All sections were imaged on a LSM 880 Airyscan confocal microscope. To avoid cross talk, Alexa Fluor 555 and Alexa Fluor 594 fluorochromes were exited at 514 and 633 nm, respectively.

### Statistics

Data are presented as mean ± SEM. Statistical analysis was performed using GraphPad Prism 6. The data were analyzed by two-sided *t* test (two groups) or one-way analysis of variance (ANOVA) followed by Bonferroni’s multiple comparison test (> 2 groups). *P* values of 0.05 or less were considered significant.

## Results

### Cardiac ischemia/reperfusion injury activates RANK-RANKL-OPG axis in mice

**Fig. 2:**
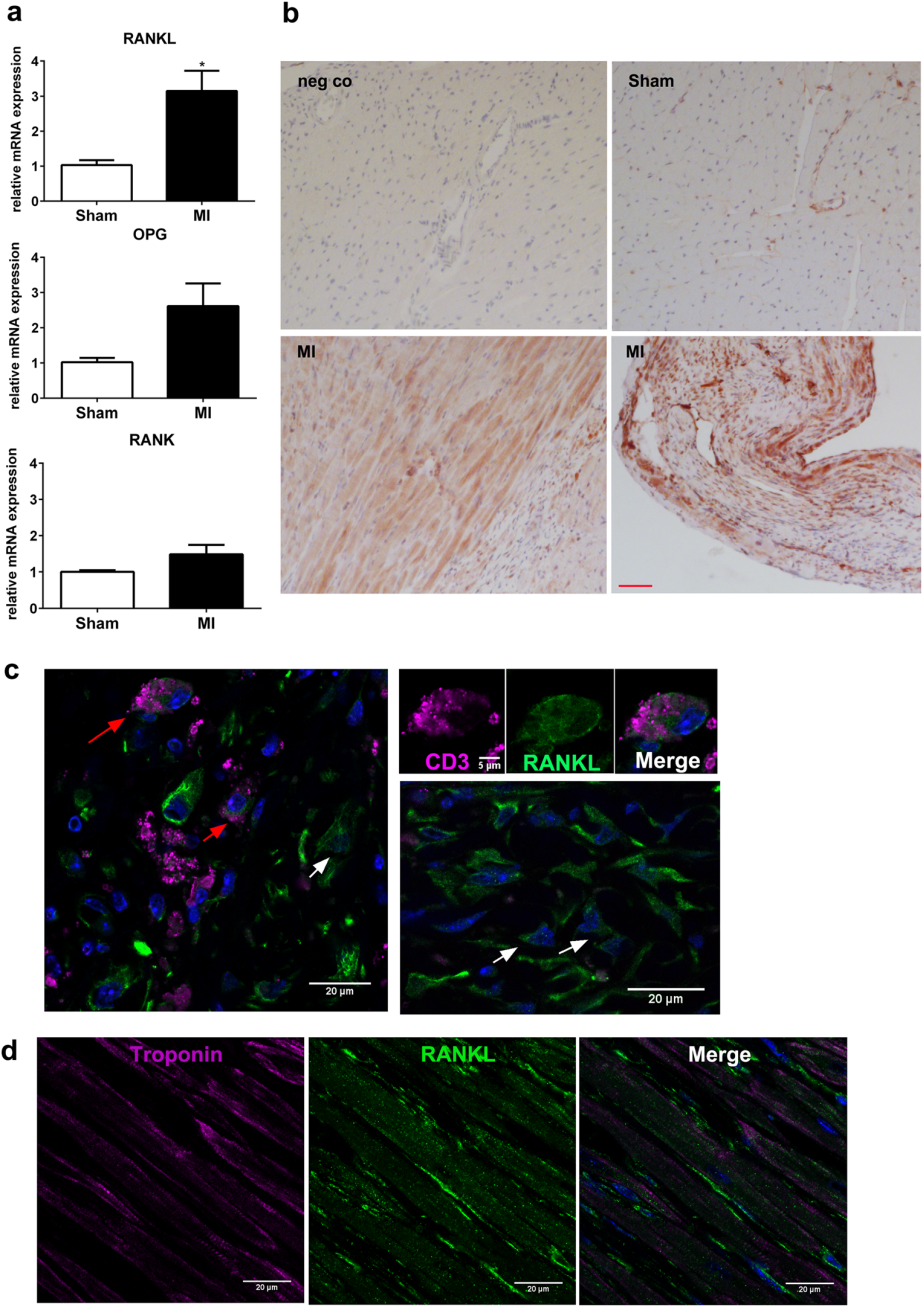
Cardiac activation of the RANK-RANKL-OPG axis after myocardial infarction (MI) in mice. **a** Gene expression analysis in the left ventricle (LV), 4 weeks after MI (*n* = 4 per group). *p < 0.05 vs. sham. **b** Immunohistochemical anti-RANKL staining in paraffin sections of the LV in sham and MI mice, 4-weeks after MI. Upper left panel: negative (neg co) performed by omitting the primary anti-RANKL antibody. Upper right panel: sham control. Lower left panel: positive RANKL staining in cardiomyocytes (CM) and infiltrating cells in peri-ischemic LV region. Lower right panel: strong RANKL staining in remaining CM and infiltrating cells of the infarcted region. Bar = 100 μm. **c** Immunofluorescent co-staining of CD3 and RANKL in cardiac paraffin sections, 4 weeks after MI. RANKL co-localizes with some CD3^+^ cells (red arrows), but is mainly expressed by CD3-negative cells with a fibroblast-like morphology (white arrows) in the infarct region. **d** Co-localization of RANKL and troponin T in the infarct border region, 4 weeks after MI. Bar = 20 μm in **c** and **d**

In line with data reported by Ueland et al. [7] in rats, we found that myocardial infarction (MI) activated the myocardial RANK/RANKL/OPG axis also in mice (Fig. 2). Left ventricular (LV) mRNA abundance of *Rankl* significantly increased, 4 weeks after MI (Fig. 2a). *OPG* and *Rank* gene expression also tended to increase in the LV after MI, but this effect did not reach statistical significance (Fig. 2a). Immunohistochemical analysis showed that RANKL expression was mainly induced in cardiomyocytes adjacent to the infarct region as well as in the cellular infiltrate within the infarct (Fig. 2b). Further analysis of the infarct region by immunofluorescent imaging revealed that RANKL co-localized with some CD3-positive T lymphocytes, but more abundant RANKL expression was present in fibroblast-like cells, cardiomyocytes, and blood vessels (Fig. 2c, d and Suppl. Fig. S1). These findings suggest that cells other than lymphocytes are the main RANKL source in the infarcted myocardium. Cardiomyocytes in remote myocardium did not express RANKL (Suppl. Fig. S1).

### Global RANKL inhibition by AMG161 lacks beneficial effect in murine myocardial infarction model

The exact pathophysiological role of increased cardiac RANKL after cardiac ischemia is not known. Because RANKL was reported to promote inflammation and matrix degradation [7, 9], we hypothesized that inhibition of RANKL could improve the post-infarct outcome after MI. To test this hypothesis, we induced MI in huRANKL-KI mice and subsequently treated the mice with the monoclonal anti-human RANKL antibody AMG161. Biological activity of AMG161 was confirmed by significantly increased femoral BMD, as well as suppressed serum alkaline phosphatase and urinary DPD excretion in AMG161-treated huRANKL-KI mice (Table 1). However, treatment with AMG161 had no effect on calcium or phosphate serum levels (Table 1).

**Table 1:** Basic characteristics, femoral BMD, blood parameters, and urinary deoxypyridinoline excretion after global RANKL inhibition in huRANKL-KI mice, 4 weeks after surgery

	Sham Co Ab	Sham AMG161	MI Co Ab	MI AMG161
Body weight (g)	29.7 ± 1.1	29.1 ± 0.7	30.6 ± 0.6	30.2 ± 0.6
Lung/body weight ratio (mg/g)	5.1 ± 0.1	5.3 ± 0.1	5.2 ± 0.2	5.0 ± 0.1
Heart/body weight ratio (mg/g)	4.1 ± 0.1	4.2 ± 0.1	4.7 ± 0.2*	4.8 ± 0.1^**#**^
Femoral metaph. total BMD (mg/cm^3^)	478 ± 4	527 ± 16*	469 ± 10	523 ± 17^§§^
Serum P (mmol/L)	2.53 ± 0.12	2.48 ± 0.21	2.94 ± 0.21	2.99 ± 0.31
Serum Ca (mmol/L)	2.11 ± 0.04	2.02 ± 0.04	2.14 ± 0.06	2.13 ± 0.05
Serum ALP (U/L)	41.1 ± 2.1	35.2 ± 3.2	41.6 ± 3.7	32.5 ± 0.8^§^
Urinary DPD/creatinine (nM/mM)	8.2 ± 1.9	1.6 ± 1.4	10.7 ± 3.5	2.9 ± 1.1^§^

*n* = 5–10 per group

*p < 0.05 vs. sham + control antibody (Co Ab)

^#^*p* < 0.05 vs. sham + AMG161

^§^*p* < 0.05 vs. MI + Co Ab

^§§^*p* < 0.01 vs. MI + Co Ab

**Fig. 3:**
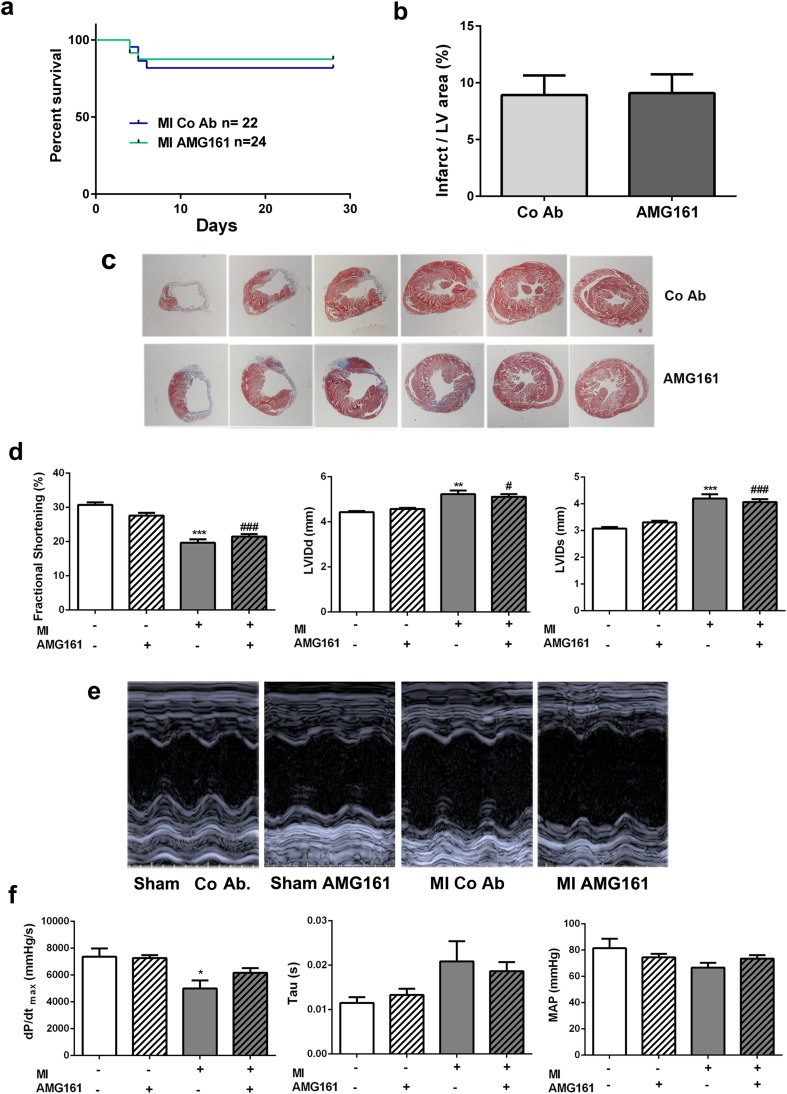
Global RANKL inhibition by AMG161 does not influence outcome in huRANKL-KI mice after MI. **a** Kaplan-Meier survival curves after MI (*n* = 22–24 per group). **b** Infarct size measured by planimetry after Masson’s trichrome staining (*n* = 11–13 per group). **c** Representative Masson’s trichrome-stained cardiac cross-sections, 4-weeks after sham or MI surgery. **d** Cardiac function and LV diameters measured by echocardiography (*n* = 10–24 per group). **e** Representative M-mode echocardiograms, obtained 3 weeks after MI. **f** Cardiac parameters measured by intra-cardiac catheterization (*n* = 5–14 per group). LVIDd left ventricle internal diameter in diastole, LVIDs left ventricle internal diameter in systole, MAP mean arterial pressure, dP/dt maximal rate of left ventricle pressure rise. *p < 0.05 vs. sham + control antibody (Co Ab); **p < 0.01 vs. sham + Co Ab; ***p < 0.001 vs. sham + Co Ab; ^#^*p* < 0.05 vs. sham + AMG161; ^###^*p* < 0.001 vs. sham + AMG161

Induction of MI led to a significant deterioration of cardiac function in huRANKL-KI mice, as evidenced by reduced fractional shortening, dilation of the LV in both systole and diastole, and diminished contractile cardiac function as assessed by dP/dt (Fig. 3). Global inhibition of RANKL after MI by treatment of huRANKL-KI mice with AMG161 did not have any statistically significant effects on survival, heart/body weight ratio, infarct size, or cardiac functional parameters compared to isotype control antibody-treated huRANKL-KI MI mice (Fig. 3 and Table 1).

### Hematopoietic, but not mesenchymal, RANKL inhibition reduced pro-inflammatory cytokine production in the left ventricle of MI mice

Depending on the cell type, RANKL can exert both pro- and anti-inflammatory effects [11, 16] and the lack of therapeutic effect of global RANKL inhibition may be caused by opposing RANKL actions in the myocardium vs. the cellular infiltrate. Thus, we next asked the question whether selective blockade of hematopoietic and mesenchymal RANKL might have positive therapeutic effects.

To selectively block hematopoietic RANKL, we lethally irradiated wt mice and reconstituted them with bone marrow from huRANKL-KI mice to replace their hematopoietic compartment with cells responsive to AMG161, using a previously established protocol [15]. To prove that the expected RANKL form was expressed by cells of hematopoietic origin after bone marrow transfer, we analyzed splenic *Rankl* gene expression in non-irradiated mice as well as after bone marrow transfer. As expected, non-irradiated wt mice did not express chimeric *Rankl*, and huRANKL-KI mice did not express wt *Rankl* in their spleens, respectively (Suppl. Fig. S2). In contrast, after lethal irradiation and vice versa reconstitution, chimeric *Rankl* gene was abundantly expressed in the spleen of reconstituted wt mice, whereas reconstituted huRANKL-KI mice expressed the mouse wt *Rankl* gene in their spleens (Suppl. Fig. S2), indicating successful exchange of the hematopoietic compartment.

**Fig. 4:**
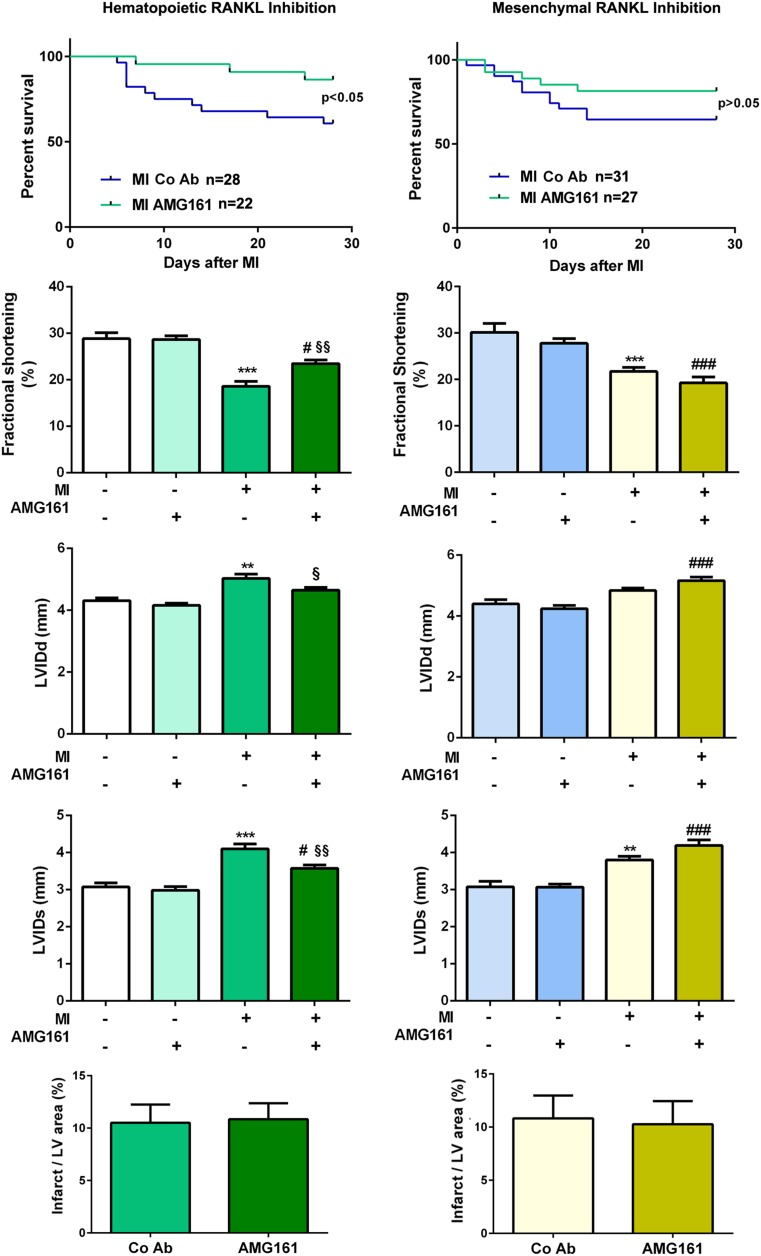
Inhibition of RANKL derived from hematopoietic (left column) but not from mesenchymal cellular sources (right column) improves survival and cardiac function post-MI. Kaplan-Meier survival curves after MI (*n* = 22–31 per group), echocardiographic parameters measured 3 weeks after surgery (*n* = 16–17 per group), and infarct size measured by planimetry after Masson’s trichrome staining (*n* = 8–14 per group) in sham and MI huRANKL-KI mice treated with AMG161 or control antibody (Co Ab), 4 weeks after MI. LVIDd left ventricular internal diameter in diastole, LVIDs left ventricular internal diameter in systole. **p < 0.01 and ***p < 0.001 vs. sham + Co Ab; ^#^*p* < 0.05 and ^###^*p* < 0.001 vs. sham + AMG161; ^§^*p* < 0.05 and ^§§^*p* < 0.01 vs. MI + Co Ab

Surprisingly, hematopoietic RANKL inhibition improved post-infarct survival as well as cardiac function as shown by a significant rise in fractional shortening, lower diastolic and systolic endocardial LV diameters, and enhanced LV contractility in AMG161 vs. control Ab-treated mice (Fig. 4 and Table 2). In contrast, inhibition of RANKL derived from the mesenchymal cell compartment in AMG161-treated huRANKL-KI mice reconstituted with wt bone marrow did not have any effect on survival or post-infarct cardiac function (Fig. 4 and Table 2). Infarct area was not affected by RANKL inhibition in any of the investigated groups (Fig. 4), suggesting that mechanisms other than those regulating cardiomyocyte cell death are responsible for the protective effects on survival and cardiac function seen after inhibition of hematopoietic cell-derived RANKL.

**Table 2:** Hemodynamic variables measured invasively using intra-cardiac catheter, 4 weeks after MI

	Hematopoietic RANKL inhibition		Mesenchymal RANKL inhibition	
	MI + Co Ab	MI + AMG161	MI + Co Ab	MI + AMG161
Systolic P (mmHg)	86.95 ± 7.5	93.18 ± 2.0	80.03 ± 3.3	84.57 ± 3.6
Diastolic P (mmHg)	60.17 ± 4.9	64.81 ± 1.7	53.87 ± 3.1	59.78 ± 4.0
MAP (mmHg)	76.57 ± 5.9	78.15 ± 1.6	66.49 ± 3.1	72.30 ± 4.0
dP/dt_max_ (mmHg/s)	4416 ± 546	6744 ± 571*	4912 ± 281	5178 ± 602
EDP (mmHg)	8.76 ± 1.9	8.19 ± 1.7	8.27 ± 2.4	9.46 ± 3.1
*Tau* (ms)	2.33 ± 0.3	1.91 ± 0.3	2.26 ± 0.4	1.78 ± 0.2

*n* = 4–6 per group

*MAP* mean arterial pressure, *dP/dt* maximal rate of left ventricle pressure rise, *EDP* end-diastolic pressure, *Tau* left ventricular relaxation time constant

*p < 0.05 vs. MI + Co Ab

**Fig. 5:**
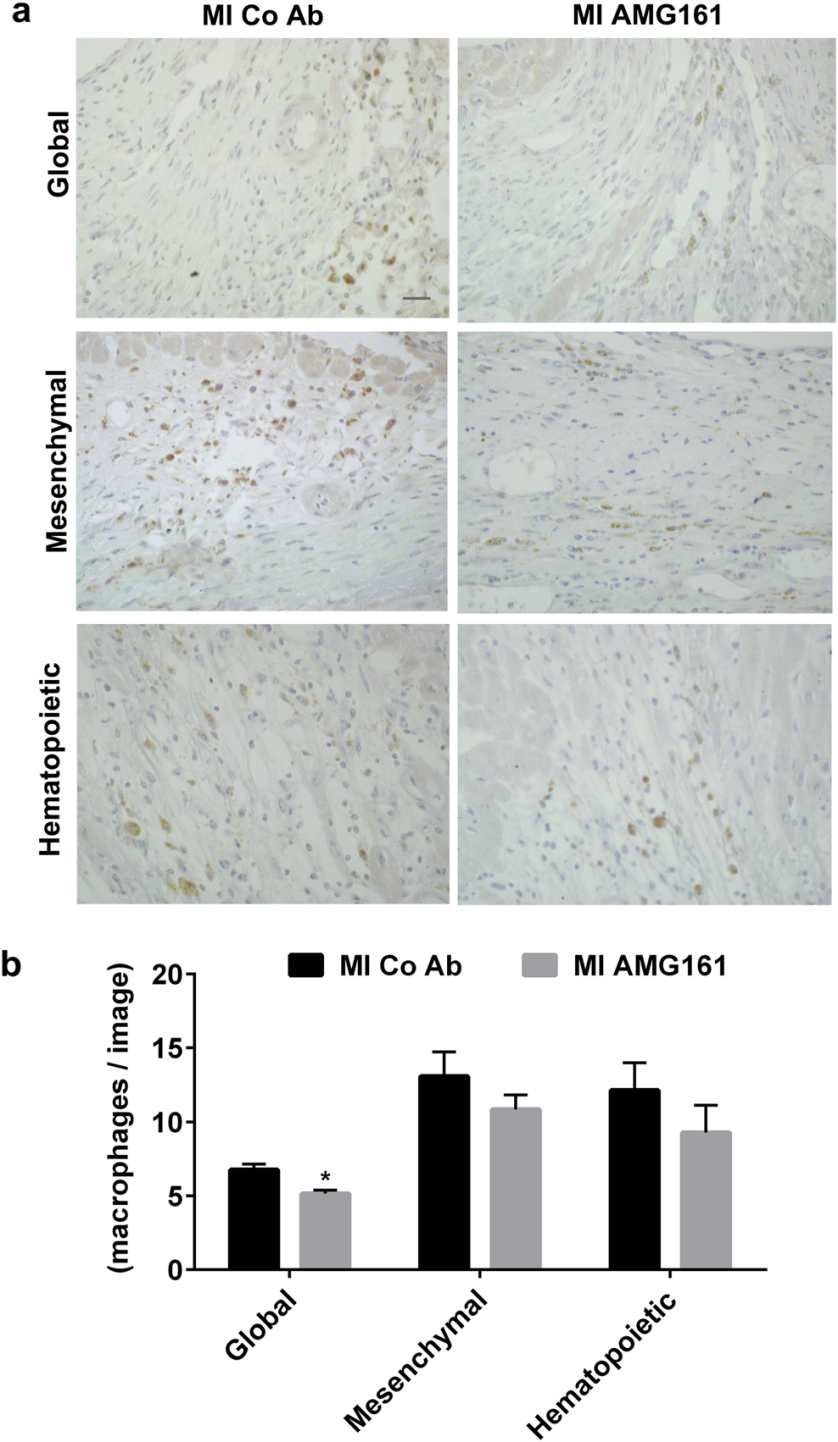
Global RANKL inhibition slightly reduces CD68^+^ macrophage abundance in the left ventricle, 4 weeks after MI. **a** Representative images of immunohistochemical stainings of CD68-expressing macrophages in the infarct region after global, mesenchymal, and hematopoietic RANKL inhibition. Bar = 50 μm. **b** Quantification of CD68^+^ macrophages in the whole infarct region, presented as cell number per image. *n* = 4–5 mice per group. *p < 0.05 vs. MI + Co Ab

**Fig. 6:**
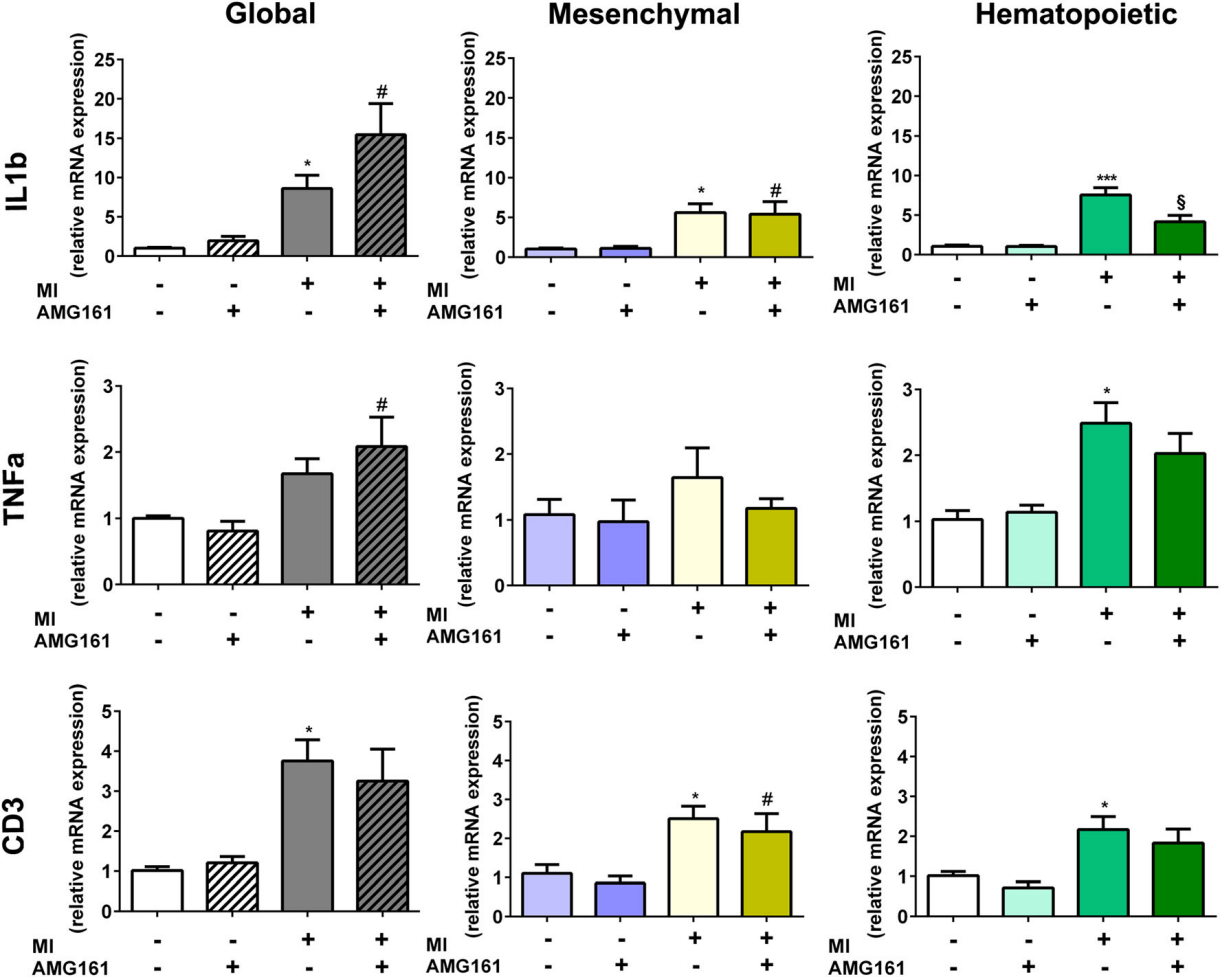
Hematopoietic but not global or mesenchymal RANKL inhibition downregulates IL-1β mRNA expression in the left ventricle after MI. Left ventricular expression of *IL-1ß* (upper panels), *TNFα* (middle panels), and *CD3* mRNA (lower panels) after global, mesenchymal, or hematopoietic RANKL inhibition. Gene expression is presented as fold increase compared to sham + control antibody (Co Ab) group. *n* = 3–9 per group. *p < 0.05 and ***p < 0.001 vs. sham + Co Ab; ^#^*p* < 0.05 vs. sham + AMG161; ^§^*p* < 0.05 vs. MI + Co Ab

To shed more light on the intriguing finding that inhibition of hematopoietic, but not of mesenchymal or global, RANKL had these beneficial effects after MI, we measured inflammatory cell infiltration and the mRNA abundance of pro-inflammatory cytokines in the LV. Because hematopoietic RANKL inhibition protected against post-ischemic LV chamber dilation, we hypothesized that inflammatory signaling pathways initiated by RANKL secreted from cells of hematopoietic origin may drive adverse post-ischemic remodeling of the LV. We first examined whether RANKL inhibition altered the post-ischemic infiltration with macrophages or lymphocytes. Immunohistochemical analysis of CD68-positive macrophages in the infarct region revealed a trend for reduced macrophage infiltration after global, mesenchymal, and hematopoietic RANKL inhibition (Fig. 5). However, this effect reached statistical significance only after global RANKL inhibition (Fig. 5). In contrast, the LV mRNA abundance of the lymphocyte-specific genes *CD3* remained unchanged after global, mesenchymal, or hematopoietic RANKL inhibition (Fig. 6). Interestingly, however, the post-ischemic rise in LV gene expression of *IL-1ß* and *Mmp-9*, but not of *TNFα*, was significantly reduced after hematopoietic RANKL blockade (Fig. 6 and Suppl. Fig. S3). In contrast, mesenchymal RANKL inhibition did not change the LV expression pattern of pro-inflammatory genes after MI, and global RANKL inhibition even enhanced *IL-1ß* mRNA expression in the post-ischemic LV (Fig. 6). Intriguingly, mRNA expression of the resolving M2 macrophage markers *Mrc-1*, *Arg-1*, and *Ym-1* was profoundly reduced in the post-ischemic LV after hematopoietic RANKL blockade, but not after global or mesenchymal RANKL inhibition (Suppl. Fig. S4). In addition, the MI-induced upregulation of *Arg-1* and *Ym-1* was lower in huRANKL-KI mice relative to mice with a wt background (Suppl. Fig. S4).

**Fig. 7:**
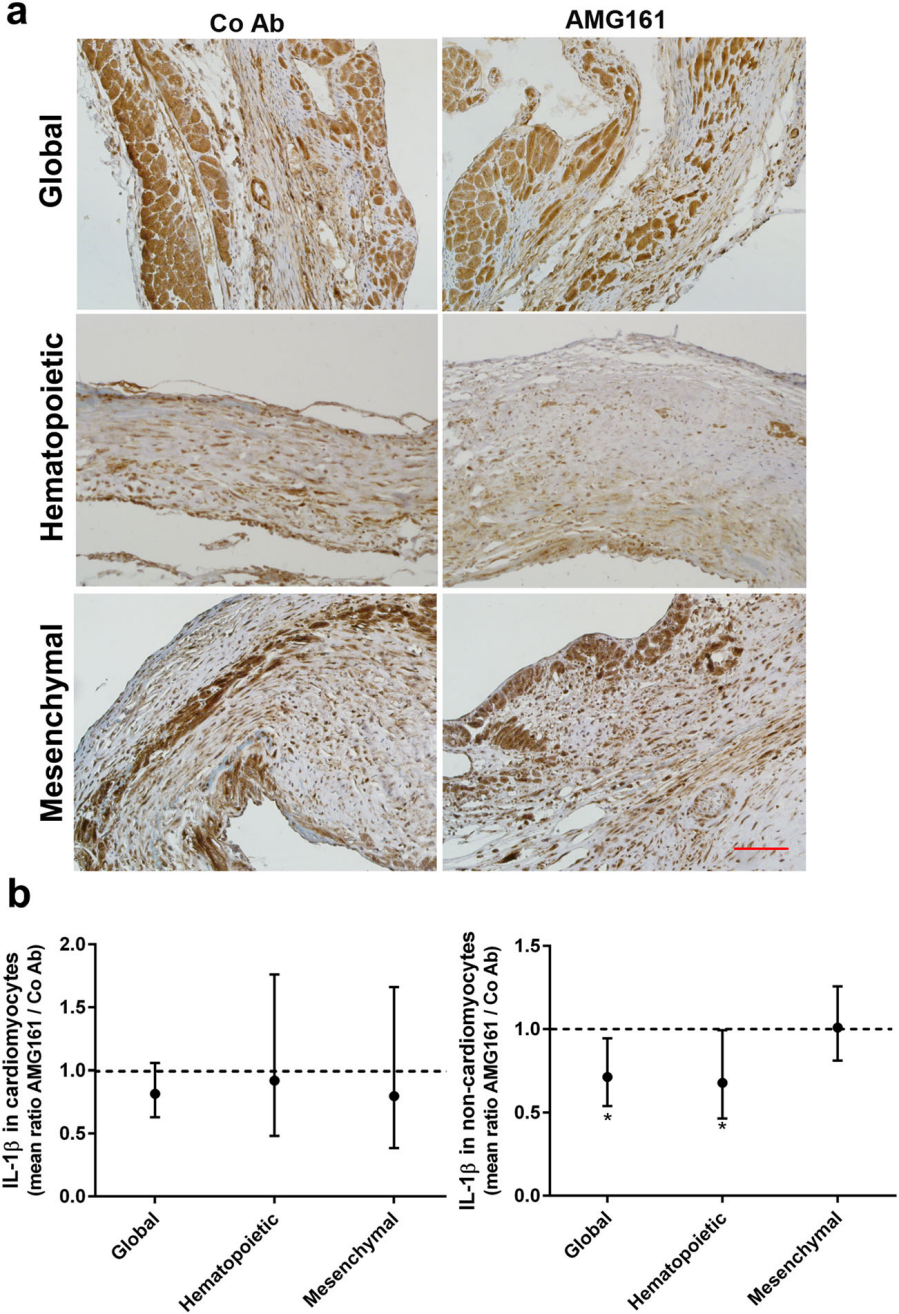
Hematopoietic and global but not mesenchymal RANKL inhibition suppresses IL-1β protein expression in infiltrating cells post-MI. **a** Representative images of immunohistochemical anti-IL-1ß staining in the infarct region, 4 weeks after MI. Bar = 100 μm. **b** Semi-quantitative analysis of IL-1ß expression in cardiomyocytes and non-cardiomyocyte cells. IL-1ß staining intensity is shown as the mean of intensity ratios between AMG161 and control antibody (Co Ab) treatment with 95% confidence intervals. Samples stained in the same experiment were paired (Co Ab and AMG161-treated). *p < 0.05 by ratio paired t-test

**Fig. 8:**
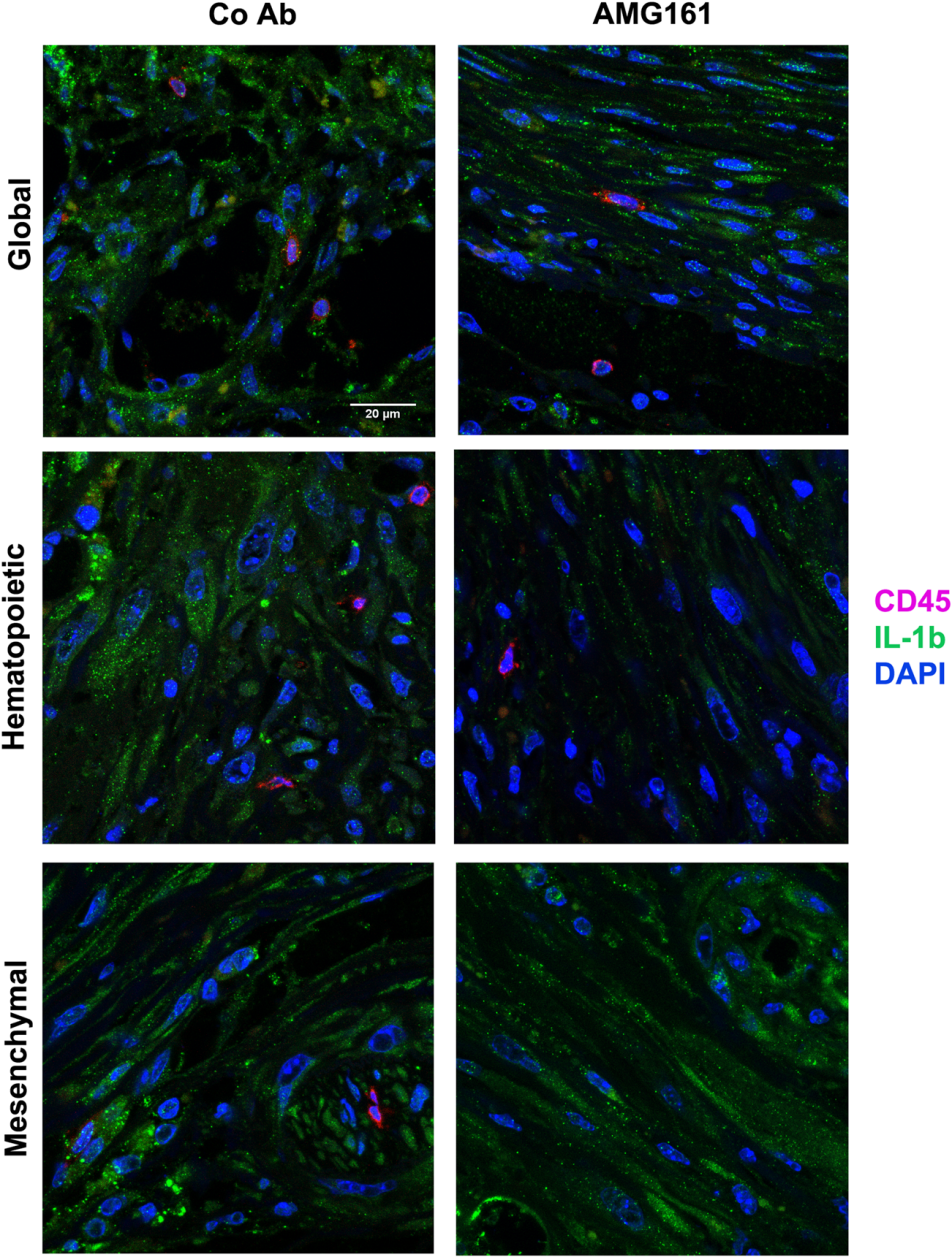
Immunofluorescent co-staining of IL-1β and CD45 in the infarct region after global, hematopoietic, and mesenchymal RANKL inhibition. IL-1β is co-expressed by some CD45^+^ cells, but abundantly expressed in fibroblast-like cells within the infarct region, 4 weeks post-MI. Note the striking reduction of IL-1β staining after hematopoietic RANKL inhibition (right middle panel). Representative images of *n* = 2 mice per group. Bar = 20 μm

To further characterize the changes in IL-1ß protein expression induced by RANKL blockade, we used immunohistochemical analysis of the infarct region. As shown in Fig. 7, inhibition of global or hematopoietic RANKL decreased IL-1ß expression in the non-cardiomyocyte cell compartment of the scar, whereas IL-1ß expression was not influenced by RANKL inhibition in surviving cardiomyocytes within the ischemic zone. To characterize further the cellular source of IL-1ß, we performed co-immunostaining of IL-1ß with CD45 and cardiac troponin T (Fig. 8 and Suppl. Fig. S5 and S6). Although some CD45^+^ cells in the infarct region expressed IL-1ß, the majority of IL-1ß-expressing cells had a fibroblast-like morphology (Fig. 8 and Suppl. Fig. S5). Troponin-positive cardiomyocytes in the border zone of the infarct also clearly expressed IL-1ß (Suppl. Fig. S6), but their IL-1ß expression, in contrast to non-cardiomyocyte cells (Fig. 8), was not downregulated by hematopoietic RANKL inhibition. Altogether, these findings suggest that RANKL derived from scar-infiltrating cells of hematopoietic origin is an important pro-inflammatory stimulus whose inhibition can be beneficial for post-ischemic recovery.

## Discussion

RANKL plays a pivotal role in bone remodeling and in immunity [3, 17, 18] and may also be an important signaling molecule in diseases affecting the cardiovascular system. We show here that experimental MI induced LV RANKL expression in mice. Global RANKL inhibition or inhibition of RANKL derived from mesenchymal cellular sources using a monoclonal anti-RANKL antibody did not significantly alter cardiac function post-MI, whereas specific inhibition of RANKL derived from hematopoietic cellular sources improved post-ischemic cardiac function, reduced mortality, and downregulated post-ischemic production of inflammatory cytokines.

In the current study, we used a monoclonal antibody AMG161 to block RANKL. Because AMG161 selectively blocks human RANKL, but not murine RANKL, we used huRANKL-KI mice expressing a humanized RANKL protein in this study. Although hematopoietic RANKL inhibition was beneficial for cardiac function after MI in WT mice, global RANKL inhibition did not significantly change cardiac function after MI in huRANKL-KI mice. Reason for this discrepancy may lay in reduced in vivo activity of chimeric RANKL in transgenic animals compared to that of murine RANKL in wt mice. Although chimeric RANKL and wt murine RANKL have the same affinity to the murine RANK receptor, the osteoclastogenic potency of chimeric RANKL was slightly lower than that of murine wt RANKL [13]. It is conceivable that the lack of myocardial MMP-9 upregulation and the reduced upregulation of some M2 macrophage markers after MI in huRANKL-KI mice may be explained by this fact. However, the promoter and splicing regions of the *Rankl* gene are intact in huRANKL-KI mice, and thus, regulation of RANKL levels during pathological conditions such as MI is expected to be comparable to that of wt mice. Another possibility for the discrepant findings after global and hematopoietic RANKL inhibition may be the fact that global RANKL inhibition was performed in non-irradiated huRANKL-KI mice, whereas hematopoietic RANKL inhibition was performed in irradiated and reconstituted mice. However, since the inflammatory response post-MI was almost identical in irradiated (mesenchymal RANKL inhibition) and non-irradiated huRANKL-KI mice (global RANKL inhibition), it is highly unlikely that this was a major influencing factor. A third possibility is that the hematopoietic output of the bone marrow is regulated by RANKL-driven osteoclastogenesis [19, 20]. Therefore, treatment of non-irradiated and irradiated huRANKL-KI mice with AMG161 may modulate inflammatory responses through inhibition of osteoclastogenesis and altered output of bone marrow-derived cells. However, we found no major and consistent differences in lymphocyte or macrophage infiltration of the infarct between huRANKL-KI and wt mice, arguing against a major difference in the bone marrow output of inflammatory/anti-inflammatory cells.

Timing of RANKL signaling and its cellular source may be important determinants of its effect during/after ischemic injury. The tissue response to increased RANKL levels during acute ischemia may be different from prolonged RANKL signaling during tissue repair and remodeling. Signaling downstream of the RANK receptor in the myocardium probably involves NF-κB [9]. NF-κB activation during acute injury may promote cell survival and suppress apoptotic signaling [8]. Indeed, models of brain and liver ischemia demonstrated an important role of *acute* RANKL signaling in cell survival and limitation of the final infarct size [11, 12]. In contrast, a recent study reported beneficial effects of RANKL inhibition during cardiac ischemia on infarct size, 24 h after reperfusion [16]. However, in the current study, infarct size after global RANKL inhibition was not changed, 4 weeks after MI. Thus, we can exclude increased vulnerability to ischemia after RANKL inhibition. On the other hand, *prolonged* NF-κB activation through RANKL may promote inflammation and adverse cardiac remodeling [8]. We hypothesize that during post-ischemic remodeling, the cellular source of RANKL determines the effect of RANKL in the ischemic myocardium. Our results support the notion that RANKL produced by cells of hematopoietic origin, but not by cardiomyocytes, contributes to maladaptive processes, deteriorating cardiac function after myocardial infarction. In this context, it is tempting to speculate that cells of hematopoietic origin, in contrast to cells of mesenchymal origin, do not produce enough OPG to inhibit excess RANKL signaling. This hypothesis is supported by findings that osteoblasts but not activated lymphocytes secrete OPG in the culture medium, and that OPG mRNA was not detected in human T lymphocytes or monocytes [5]. Hence, the local increase in RANKL secretion from BM-derived cells may be a driving force of inflammation in the heart after MI, whereas RANKL produced by cardiomyocytes may be inhibited by concurrently increased cardiomyocytic OPG secretion. This notion may explain why inhibition of hematopoietic RANKL has beneficial effects post-MI, whereas selective inhibition of mesenchymal RANKL does not influence post-ischemic cardiac remodeling.

A puzzling observation in our study was that post-ischemic LV *IL-1ß* mRNA abundance was promoted after global RANKL inhibition, but reduced when only hematopoietic RANKL was inhibited. There are several possible explanations for this finding. Global RANKL inhibition using AMG161 may leave larger amounts of OPG available for other signaling pathways, including binding of TRAIL. Furthermore, it was reported that RANKL may actually reduce both innate [11, 21] and adaptive immune responses [22] in some models. For example, it has been shown that RANKL expression in keratinocytes can drive formation of regulatory T cells [22]. In analogy, RANKL expression on cardiomyocytes may downregulate the inflammatory response post-MI. However, since inhibition of mesenchymal RANKL had no influence on LV function or gene expression, it is unlikely that the latter scenario is true. It may also be important to note in this context that the increased LV *IL-1ß* mRNA expression observed after global RANKL inhibition in huRANKL-KI MI mice was not evident in immunohistochemical analyses of the scar region (Figs. 7 and 8). Therefore, it is unclear whether the increased *IL-1ß* gene transcription after global RANKL inhibition fully translates into augmented IL-1ß secretion at the protein level. On the other hand, the downregulation of *IL-1ß* mRNA abundance in the LV after hematopoietic RANKL inhibition was confirmed at the protein level by immunohistochemistry.

Secreted IL-1β has a critical role in the post-ischemic remodeling by stimulating inflammatory cell accumulation, inflammatory cytokine production, myofibroblast differentiation, extracellular matrix degradation, and collagen production [23, 24]. Moreover, IL-1β depresses cardiac contractility by reducing L-type Ca^2+^ currents in neonatal and adult ventricular cardiomyocytes [25], and by inhibiting their β-adrenergic response [26]. We show here that inhibition of hematopoietic RANKL reduces transcription of the pro-inflammatory cytokine IL-1β in the myocardium, and more specifically in scar-infiltrating inflammatory cells. This is in line with our finding of improved cardiac function in mice with reduced IL-1β levels after hematopoietic RANKL blockade.

RANKL signaling receptor RANK is present in both neutrophils [27] and macrophages [28, 29], which makes both cellular population responsive to RANKL. Role of RANKL in neutrophil infiltration and MMP-9 secretion was recently demonstrated early after MI [16]. However, in our long-term study, it is more probable that beneficial effects seen after hematopoietic RANKL inhibition are mediated by macrophages which infiltrate ischemic myocardium long term after MI [30]. Although we did not observe changes in macrophage number in the infarct region after hematopoietic RANKL blockade, markers of the reparative M2 subset were profoundly reduced in the myocardium after hematopoietic blockade of RANKL, 4 weeks post-MI. This finding may suggest that inhibition of hematopoietic RANKL may alter the time course of resolution of inflammation. Further experiments are needed to address the question of how RANKL inhibition affects resolution of inflammation after MI.

Our finding that global RANKL inhibition did not increase infarct size or deteriorate cardiac function after MI may have important clinical implications. RANKL is the molecular target behind one of the most effective osteoporosis treatments today, the treatment with the anti-RANKL antibody denosumab. The typical osteoporosis patient is of advanced age and usually at high risk to suffer co-morbidities, and there is a rising awareness that cardiovascular diseases and osteoporosis might be pathophysiologically linked diseases. A large 3-year placebo-controlled trial of denosumab in postmenopausal women with low bone mass showed no treatment-related effects on the incidence of cardiovascular events, coronary heart disease, or atrial fibrillation, with a trend towards reduced all-cause mortality in the denosumab arm [31]. However, this clinical trial population, postmenopausal women, was not selected for increased CV risk factors, and it may therefore be reassuring that the current study showed no untoward effects of global RANKL inhibition on post-MI survival or cardiac function.

Our study has shown that the upregulation of RANKL in the post-ischemic myocardium mainly involves fibroblast-like cells, blood vessels, and surviving cardiomyocytes. We and others showed earlier that a substantial amount of endothelial cells and fibroblasts/myofibroblasts in the heart is donor-derived after bone marrow transplantation [14, 32]. Therefore, it is conceivable that some beneficial effects after hematopoietic RANKL blockade are attributable to endothelial RANKL blockade. Future studies need to address the question how the availability of RANKL/RANK molecules on specific cell types regulates cell-cell interactions and the immune/inflammatory tissue response in the course of tissue repair after MI. Improved insight into these mechanisms may eventually open up new possibilities for the treatment of MI patients.

## Electronic supplementary material

ESM 1 (PDF 2140 kb)
