# Supplementary material for: Selective inhibition of receptor activator of NF-κB ligand (RANKL) in hematopoietic cells improves outcome after experimental myocardial infarction
Source: J Mol Med (Berl). 2018 May 8;96(6):559–73. doi: 10.1007/s00109-018-1641-x (PMC5988763; doi:10.1007/s00109-018-1641-x)
Supplement: Supplementary file 1 — (PDF 2140 kb) [file 109_2018_1641_MOESM1_ESM.pdf]

Supplemental material

Selective inhibition of receptor activator of NF-κB ligand (RANKL) in hematopoietic cells improves outcome after experimental myocardial infarction

Journal of Molecular Medicine

Svetlana Slavic[1], Olena Andrukhova[1], Kristopher Ford[1], Stephan Handschuh[2], Nejla Latic[1], Ursula Reichart[2], Soleman Sasgary[1], Claudia Bergow[1], Lorenz C. Hofbauer[3], Paul J. Kostenuik[4 ¶], and Reinhold G. Erben[1]

[1]Department of Biomedical Research, University of Veterinary Medicine Vienna, Vienna, Austria
[2]VetCore, University of Veterinary Medicine Vienna, Vienna, Austria
[3]Division of Endocrinology, Diabetes, and Bone Diseases, Department of Medicine III and Center for Healthy Aging, Technische Universität Dresden, Dresden, Germany
[4]Amgen Inc., Thousand Oaks, CA, USA ¶ Current address: Phylon Pharma Services, Newbury Park, CA, USA

Reinhold.Erben@vetmeduni.ac.at

Suppl. Fig. 1

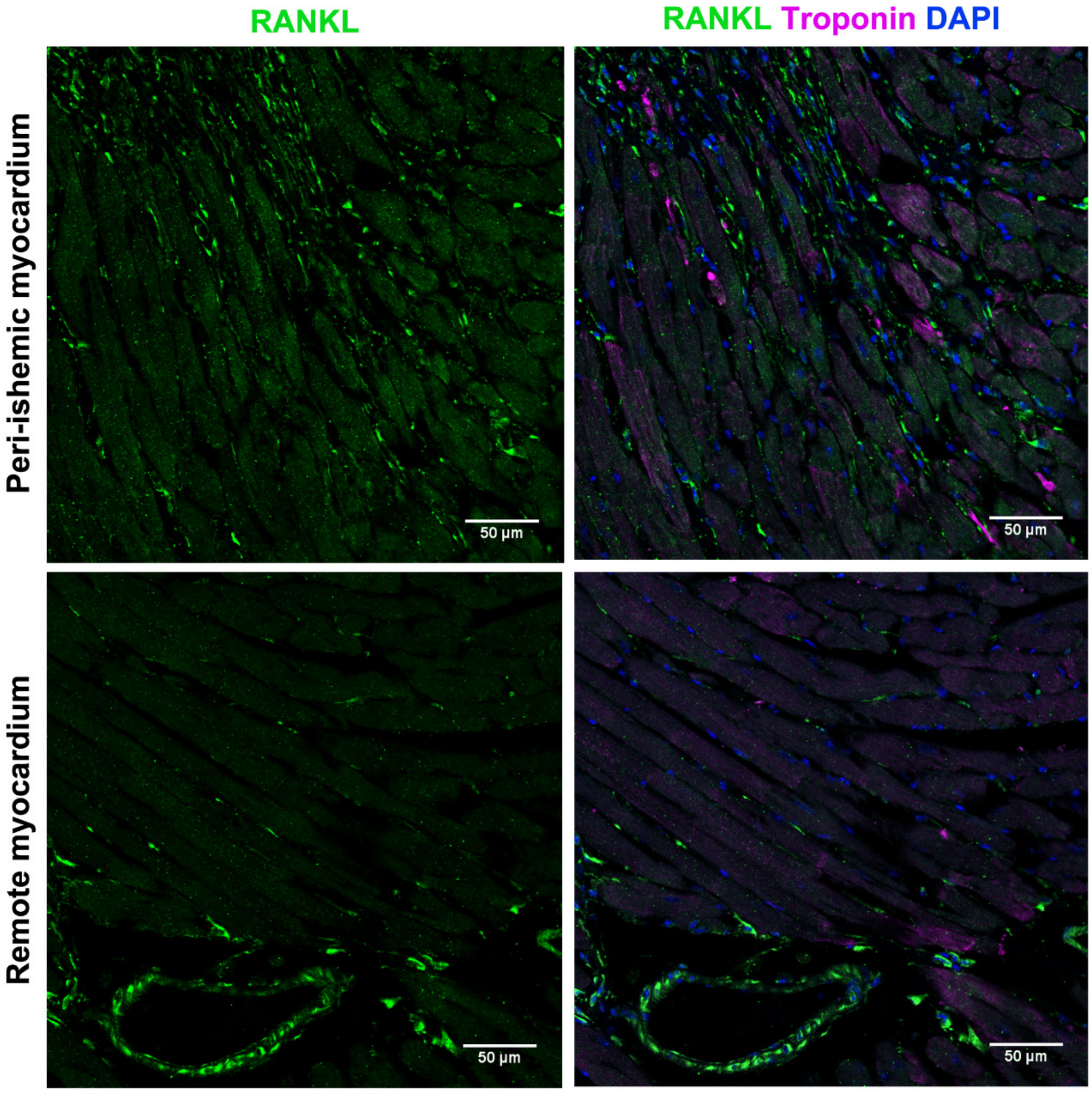

Fig. S1. Representative images of immunofluorescent co-staining of RANKL and troponin T in the peri-ischemic myocardial region (upper panels) and remote myocardium (lower panels). RANKL expression in cardiomyocytes is restricted to the peri-infarct region, whereas RANKL is strongly expressed in blood vessels in both the peri-ischemic region and remote myocardium. Bar = 50 µm.

Suppl. Fig. 2

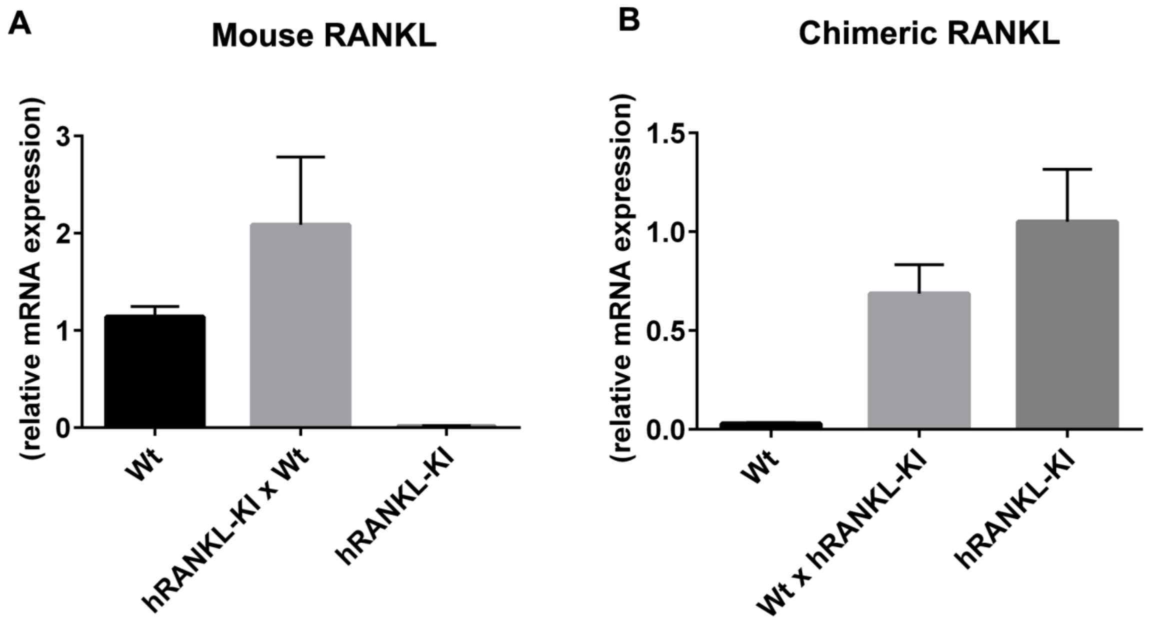

Fig. S2. mRNA expression of mouse and chimeric *Rankl* gene in the spleen quantified by qRT-PCR. **a** Non-irradiated wild-type (Wt) and huRANKL-KI mice after lethal irradiation and reconstitution with bone marrow from Wt donors (hRANKL-KI x Wt) express comparable levels of wt mouse *Rankl* gene, whereas murine *Rankl* is undetectable in the spleen of non-irradiated huRANKL-KI mice,. **b** Wt mice after lethal irradiation and reconstitution with bone marrow from huRANKL-KI donors (Wt x hRANKL-KI) express similar levels of chimeric *Rankl* gene as non-irradiated huRANKL-KI mice. Chimeric *Rankl* is undetectable in non-irradiated Wt mice. n=7-9 per group.

Suppl. Fig. 3

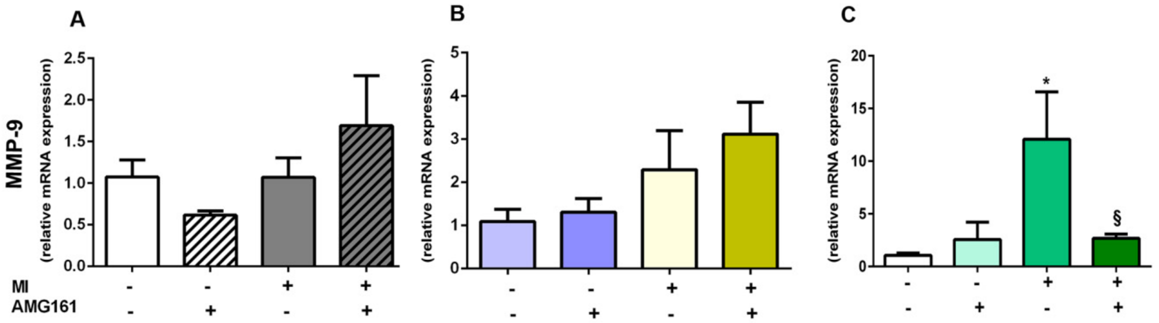

Fig. S3. mRNA expression of MMP-9 in the left ventricle, 4 weeks after MI. **a** Global RANKL inhibition, **b** mesenchymal RANKL inhibition, **c** hematopoietic RANKL inhibition, n=4-9 per group; *$p < 0.05$ vs. sham + control antibody; $^{\S}p < 0.05$ vs. MI + control antibody

Suppl. Fig. 4

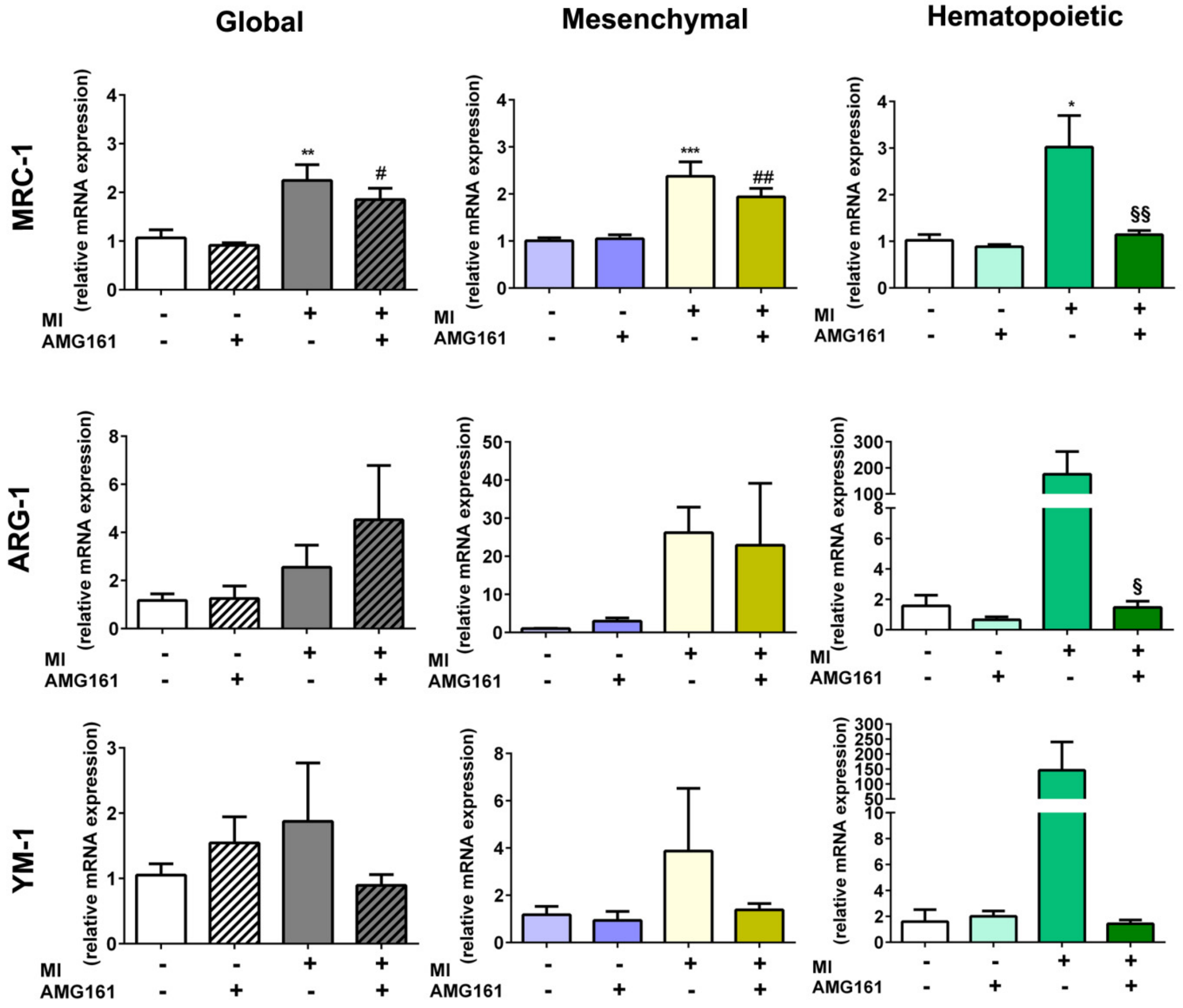

Fig. S4. mRNA expression of the M2 macrophage markers *Mrc-1*, *Arg-1* and *Ym-1* in the left ventricle, 4 weeks after MI. Relative gene expression is presented as fold increase compared to the sham + control antibody (Co Ab) group. n=3-9 per group, *p < 0.05, **p<0.01 and ***p < 0.001 vs. sham + Co Ab; ##p<0.01 vs. sham + AMG161; §p < 0.05 and §§p<0.01 vs. MI + Co Ab.

Suppl. Fig. 5

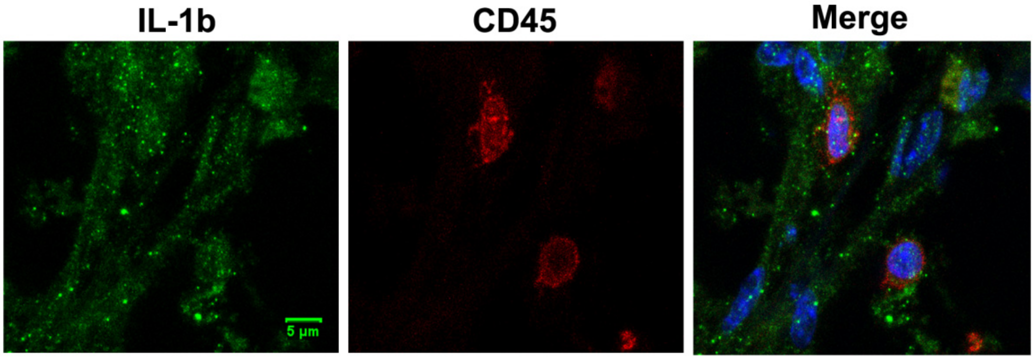

Fig. S5. Confocal images of fluorescent co-staining of Il-1β (green) and CD45 (red) in the infarct region, 4 weeks post-MI. Bar = 5 µm.

Suppl. Fig. 6

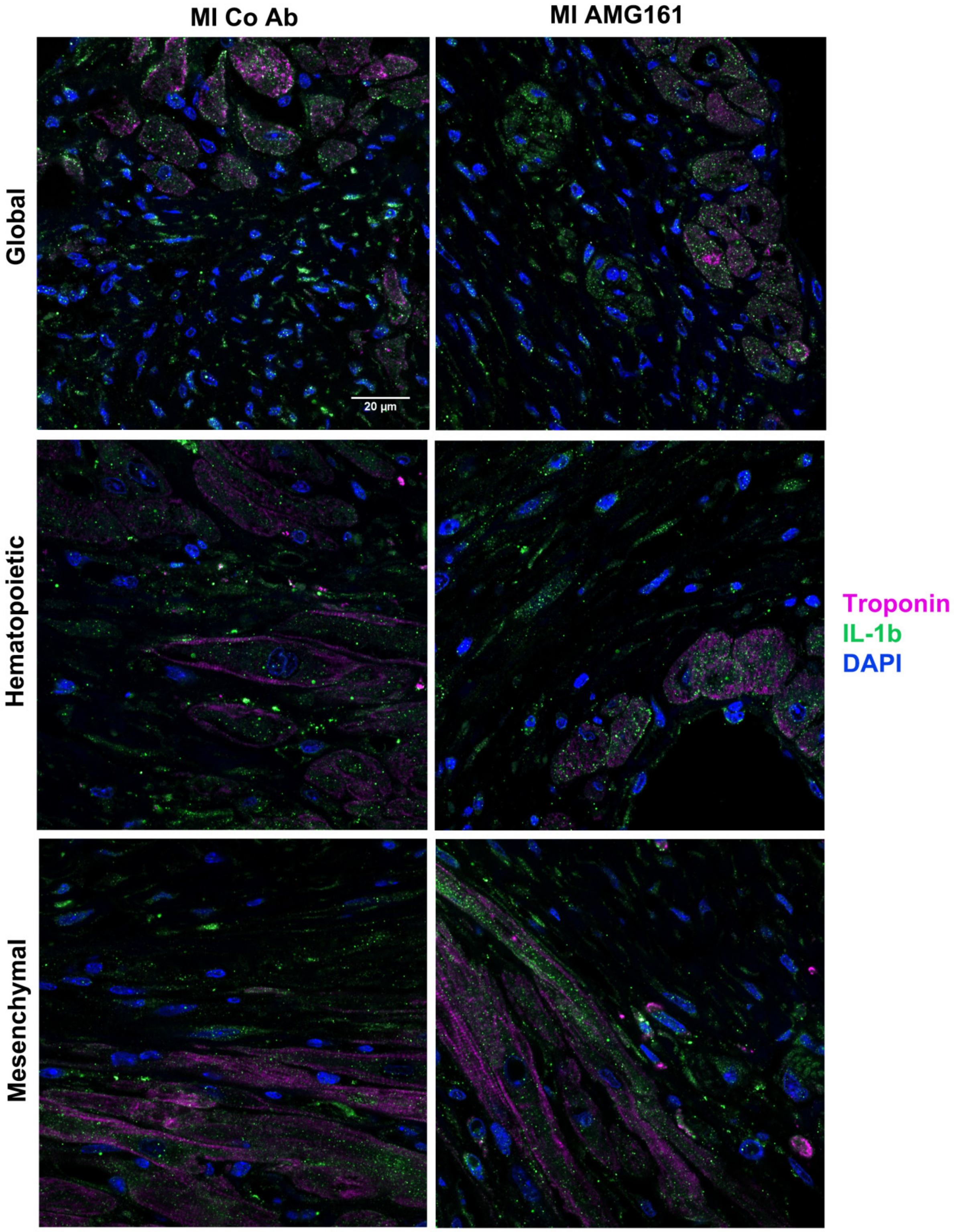

Fig. S6. Representative confocal images of Il-1ß and cardiac troponin T co-staining (n=2 mice per group) in the border zone of the infarcted region, 4 weeks after MI. Co-localisation of IL-1ß and troponin T is similar after global, hematopoietic, and mesenchymal RANKL inhibition. Bar = 20 µm.
